# Psychometric properties and measurement invariance across gender of the Italian version of the tempest self-regulation questionnaire for eating adapted for young adults

**DOI:** 10.3389/fpsyg.2022.941784

**Published:** 2022-08-18

**Authors:** Pierluigi Diotaiuti, Laura Girelli, Stefania Mancone, Giuseppe Valente, Fernando Bellizzi, Francesco Misiti, Elisa Cavicchiolo

**Affiliations:** ^1^Department of Human Sciences, Society, and Health, University of Cassino and Southern Lazio, Cassino, Italy; ^2^Department of Human, Philosophical, and Educational Sciences, University of Salerno, Fisciano, Italy

**Keywords:** eating self-regulation, temptations, goals, young adults, measurement, questionnaire, diet, TESQ-E

## Abstract

The prevalence of overweight and obesity in young adults has increased dramatically in recent decades. The unhealthy eating habits that develop at this time can often lead to negative health consequences in the future. It is therefore important to learn about self-regulation and self-control strategies and help young adults to have healthy eating behaviours. This study aims to present an Italian version of the Tempest Self-Regulation Questionnaire for Eating (TESQ-E) adapted for young adults. The instrument assesses self-regulation and self-control strategies to counteract the desire and temptation to eat unhealthy food and to choose healthy foods. A total of 645 students (271 males and 374 females with an average age of 24.82 and *SD* = 4.34) took part in the study. The results of the confirmatory factor analysis supported the six first-order factors model concerning specific self-regulation strategies and a higher-order structure of the TESQ-E [χ^2^ (152) = 336.480, *p* < 0.001; CFI = 0.95; RMSEA = 0.04; SRMR = 0.04]: three correlated second-order factors (addressing the temptation directly, addressing the psychological meaning of temptation, and addressing the goal directly) and one-third-order factor (self-regulatory competence). The results also confirmed the strong measurement invariance of the TESQ-E across gender. To have reliable comparisons across males and females, the latent mean differences tests were performed on the six specific self-regulation strategies. The analysis showed that females appear to prefer strategies that directly address the goal by expressing explicit intentions or plans to eat in a healthy way. Convergence validity was tested through correlations with Eating-Self-Efficacy Brief Scale (ESEBS), Emotional Eating Scale (EES), Scale of Attitudes towards Healthy Eating (SAHE), and *Body Mass Index* (BMI). In conclusion, the TESQ-E appears to be a psychometrically sound questionnaire that can be effectively used with young adults to measure self-regulation strategies in eating in order to plan personalised interventions for the prevention and control of the metabolic syndrome, and to reduce a wide range of diet-related diseases.

## Introduction

The TESQ-E questionnaire was developed as part of the European research project TEMPEST, which ran from 2009 to 2013 and involved nine European countries: Belgium, Denmark, Finland, Germany, the Netherlands, Poland, Portugal, Romania, and the United Kingdom. TEMPEST stands for Temptations to Eat Moderated by Personal and Environmental Self-Regulatory Tools (cordis.eu, 2013). The aim of the project was to find out how the 15,000 participating adolescents (10–17 years old) could learn to regulate their food intake in a food-filled environment.

The TEMPEST Self-Regulation Questionnaire for Eating (TESQ-E) is an instrument to investigate adolescents’ self-regulation and self-control strategies to counteract the desire and temptation to eat unhealthy food and to choose healthy foods (De Vet et al., 2014). The World Health Organisation (WHO, 2020) had already raised the alarm about the problem of obesity in the world population in the 1990s, and more recently has focused on childhood and youth obesity. The TEMPEST project was one of the campaigns to raise awareness of obesity, since obesity is recognised as a “social and environmental disease” that has repercussions not only for the health of the individual but also for the health system (Branca et al., 2007). From the point of view of risk factors, dietary style has been indicated as one of the main modifiable determinants of chronic diseases (Ezzati et al., 2004; Allison et al., 2008). Alterations in diet have, in fact, a strong impact, both positive and negative, on health throughout life.

Changes in diet can affect not only an individual’s current health, but also the likelihood of developing diseases such as cancer, cardiovascular disease, and diabetes later in life (Ness, 2004). Obesity in children and young people is associated with increased hypertension, hyperlipidaemia, type 2 diabetes, and early development of atherosclerotic lesions (Roh et al., 2007; Ahmad et al., 2010; Hwang et al., 2010; Güngör, 2014). The presence of these vascular risk factors, although not necessarily resulting in morbidity at a developmental age, increases the risk of cardiovascular disease in adulthood (Jolliffe and Janssen, 2006).

Obesity and eating behaviour can be influenced by factors both internal and external to the individual. Internal factors are determined by the biological, neurophysiological, and genetic characteristics of the individual; external, or environmental factors that affect the psychology of the individual relate to both the eating behaviour taught by parents, but also what we learn from the eating behaviour we see implemented by others (Herman, 2015; Hughes and Frazier-Wood, 2016).

Another very important external factor is related to the choices of the food industry and concerns the availability of quality food. In fact, the food industry, through particular food processing and marketing actions, encourages people to consume products, whose main characteristic becomes desirability, often at the expense of quality; thus foods that combine fat and carbohydrates tend to be preferred by individuals as they stimulate the areas of the brain related to gratification and reward (DiFeliceantonio et al., 2018).

Some individuals may have an inherited genetic predisposition (Vainik et al., 2019) that influences the ability to self-regulate (Rankinen and Bouchard, 2006) through various neurological systems, including for example the dopaminergic system (Kirschner et al., 2020): a 2-year-old child with good self-regulatory ability is likely to have a lower BMI and lower risk of obesity at age 5 (Graziano et al., 2010), just as young people who have a greater ability to delay gratification have lower levels of overweight and slower weight gain in childhood (Francis and Susman, 2009; Seeyave et al., 2009; Duckworth et al., 2010).

Eating behaviours acquired in adolescence are usually maintained as adults (Dietz, 1997) and become resistant to change (Lytle et al., 2000; Lien et al., 2001) and therefore may have an impact on disease prevention in the medium and long term (Altekruse et al., 1997). Food consumption patterns in adolescence are significantly influenced by the cultural dimension which, starting with the influence of the family context (Benton, 2004; Verzeletti et al., 2010), is also increasingly linked to that of the peer group and society (Salvy et al., 2009).

However, entering adulthood is a key phase for health. The unhealthy eating behaviour that develops at this time can often lead to negative health consequences in the future. The rates of overweight and obesity have increased dramatically in recent decades (Nelson et al., 2008; Mistretta et al., 2017), and the prevalence of overweight and obesity is higher in young adults than in other age groups (Poobalan et al., 2014). Young adults leaving the family home often have no correct eating habits. Moreover, a fast pace of life, professional work, studies, time constraints and lack of adequate financial resources, as well as stress and distress are the reasons why the “fast food” menu dominates the diet of young adults (Mattsson and Helmersson, 2007; Harris et al., 2010; Bugge, 2011). Furthermore, eating out means that the servings contain less fibre and more calories, saturated fats and cholesterol, which negatively affects the body composition of consumers (Bergman et al., 2021). Another problem is skipping breakfast or too low consumption of vegetables and fruit (Larson et al., 2007). Meldrum et al. (2017) indicates that the lack of diversity in nutrition, insufficient number of meals and their irregularity, as well as snacking between meals is also a significant problem.

A large proportion of young people have therefore a food consumption profile based on the following criteria: simplicity, speed, and convenience in food preparation, and a non-conventional form of consumption, i.e., they combine the act of eating with other types of activities (TV, radio, reading, etc.; Sanlier et al., 2018; Kartal and Ayhan, 2020). A better understanding of the motivations and barriers to healthier eating habits in young adults is important for the development of behavioural change programs and strategies to improve lifestyle in general and, in particular, to reduce diet-related diseases.

Several studies show that some people are more successful in food control while others do not succeed in implementing planned but rather uninhibited eating behaviours (Rankinen and Bouchard, 2006; Kuijer et al., 2008; Papies et al., 2008). Therefore, it is important to learn about self-regulation and self-control strategies from a young age and it is useful to help people to have a healthy eating behaviour as adults.

The TESQ-E instrument measures self-regulation on the basis of established theoretical models of behaviour and the psychological meaning attached to both temptations and goals (e.g., Alivernini et al., 2018) that an individual wishes to achieve. The instrument was constructed by exploring the strategies implemented spontaneously by the adolescents and then testing against the available test batteries for each strategy. The aim was to develop a single instrument capable of giving a reliable measure of dietary self-regulation. The instrument allows the identification of six strategies used by adolescents to self-regulate and control themselves (e.g., Alivernini et al., 2008; Girelli et al., 2018), which is a fundamental skill for healthy eating behaviour as adults (Wills et al., 2007; Gerrits et al., 2010). The strategies are, respectively: avoiding temptation; controlling temptation; distraction; suppression; goal and rule setting; and goal deliberation. In the original model, each strategy consists of four items.

In Italy, over 10% of the adult population is obese and over a third is overweight with similar numbers among children (Lauro et al., 2019; Serra et al., 2020). According to the latest *Italian Obesity Barometer Report* (2020), Italy, despite having lower levels of obesity and overweight than other European countries, has seen a 30 percent increase in the incidence of overweight and obesity over the past 30 years, of which only one-third can be attributed to an ageing population. Time comparisons, analysed by gender and age group among adults, showed specific increases, particularly among women aged 18–45 (on average + 15 percent) and for people aged 75 and over in both genders, where for men there is even a 20 percent increase. For obesity, gender differences became slightly more pronounced over time, as the increase affected men to a greater extent, especially from the age of 55. The report showed also a link with influential aspects, such as the relationship with food or the adoption of dietary patterns and lifestyles acquired and ingrained in the area of birth before becoming an adult, such that in North-Central regions the prevalence of excess weight of people born in the South is higher than the regional average rate. Further recent studies reported the situation has been further complicated by the impact of the COVID-19 pandemic on the territory as a result of the restrictions suffered due to the lockdown, given also the inability to exercise in dedicated facilities and the concomitant incidence of improper, high-calorie eating, so much so that specialists have estimated an increase in at least 30 percent in eating disorders, including obesity (Pellegrini et al., 2020; Caretto et al., 2022).

Given that the regulation of dietary behaviour has become for all intents and purposes a relevant issue for the Italian context, on par with other European states, this study aims to present an Italian version of the TESQ-E tool adapted for young adults. As the above-mentioned contributions have shown that dietary dysregulation is also a pervasive feature of the eating behaviour of young adults, this study analysed the psychometric properties and the measurement invariance across gender of the TESQ-E. To the best of our knowledge, no study has yet proved the measurement invariance of the scale across gender and this is crucial in order to make meaningful comparisons between the different self-regulation and self-control strategies of young boys and girls. Indeed, there is evidence that supports the fact that there are gender differences associated with eating behaviour (Mond and Arrighi, 2011; Oncini and Guetto, 2018; Lombardo et al., 2019). When selecting food, women are mainly guided by dietary considerations and psychological and cultural factors related to body perception, whereas men tend to behave in a somewhat less rational and critical manner, choosing food on the basis of personal taste or acquired habits. The function attributed to food and the act of eating is also gendered: women are more often subject to craving, the intense desire for a particular food with a preference for sweet foods, also as an anti-stress function, but tend to experience hunger attacks with feelings of guilt; for men, on the other hand, the occasional binge is often associated with a positive mood (Hallam et al., 2016; Opwis et al., 2017; Taş Torun et al., 2021). In the light of this evidence, in the present study we analysed the differences in self-regulation strategies between male and female young people.

Convergent validity was assessed using several tools adopted in previous research on eating behaviour and individual responses: Eating-Self-Efficacy Brief Scale (ESEBS), Emotional Eating Scale (EES), Scale of Attitudes towards Healthy Eating (SAHE), and BMI. With reference to previous literature, we hypothesized a positive association of the strategies addressing temptations directly (Avoidance and Controlling) with Eating-Self-Efficacy (ESEBS), especially in social circumstances; a negative association of the strategies addressing the psychological meaning of eating (Distraction and Suppression) with Emotional Eating (EES); a positive association of the strategies addressing the goal directly (Goal and rule setting; Goal deliberation) with Healthy Eating Attitudes (SAHE) and the Body Mass Index (BMI).

## Materials and methods

### Linguistic procedures

The translation of the TESQ-E followed forward and backward translations of the original scale, according to the translation guidelines indicated by Beaton et al. (2000). Two Italian translators independently completed the forward translation and negotiated any differences in the two versions. The reconciled Italian version was then given to two English translators, who independently back-translated the measure. Any discrepancies were discussed and resolved, and modifications were made in the TESQ-E to take into account any rewording to improve the conceptual relevance and comprehension of the items. Finally, a small focus group of 10 components was convened and structured so that three different age groups (20–30; 31–40; and 41–50), both genders and subjects with low-medium and high educational qualifications, were represented within it. The discussion held on each item after the administration of the TESQ-E scale did not reveal problems of comprehensibility or literacy discrepancies.

### Participants and administration

For the present study, the sample size planning was based on the ability to verify an adequate fit of TESQ-E starting with a translation of the full English version, which included 24 items. Using the root-mean-square error of approximation (RMSEA) as the measure of model fit, a minimum of 240 participants provides a 90% power level to test RMSEA ≤ 0.05 when RMSEA = 0.08, using a 0.05 significance level (MacCallum et al., 1996). Participants were recruited by forwarding an email contact to students enrolled in a university in central-southern Italy. This email defined the goals as well as the function of the study. Participants were invited to enter a specific link found in the same notice, after which they filled in and posted the answers telematically and digitally. Participants were assured anonymity and also the use of information in aggregate type for research purposes. Data were collected on line using QuestBase platform (Fidenia srl). A total of 2,300 contact emails were sent out. As far as the drop-out ratio is concerned, 112 participants dropped out after beginning to fill it in, therefore 645 students (271 males and 374 females with an average age of 24.82 and *SD* = 4.34; min_age_ = 20; max_age_ = 40) completed the questionnaires. The convergent validity was tested using an additional convenient sample of participants, recruited online as well, consisting of a total 335 young people (80% females, *M*_age_ = 25.04; *SD*_age_ = 4.39; min_age_ = 20; max_age_ = 40). In order to recruit this sample, a total of 1,000 contact emails were sent out. In this case, the inclusion criteria were being enrolled in the University and the non-participation in the previous administration. The recruitment phase was carried out in the months of May and July 2021.

### Measures

#### Self-regulation questionnaire for eating

*The Self-Regulation Questionnaire for Eating* (TESQ-E) is a cross-cultural instrument that measures self-regulation in the area of eating behaviour in children and adolescents. The original version was created in English within the TEMPEST consortium^1^ and each country translated and back-translated the items into the country’s native language. The final version has 24 items, which are to be answered using a five-point scale, ranging from 1 (“never”) to 5 (“always”; De Vet et al., 2014). The instrument is organised in six self-regulation strategies [first-order factors: avoidance of temptations (four items); controlling temptations (four items); distraction (four items); suppression (four items); goal and rule setting (four items); and goal deliberation (four items)] which loaded on three general self-regulation approaches (second-order factors: strategies that directly address temptations; strategies that address the meaning of temptations and strategies that directly address the objectives; Stok et al., 2013). The first approach, temptation reduction, reflects strategies for dealing with the food environment directly and includes items that describe the control of temptation (e.g.: Ensuring that crisps are out of sight, while watching TV) and the avoidance of temptation (for example: Avoiding the candy aisle in the supermarket). The second approach, change in the meaning attributed to temptations, reflects strategies to change the meaning of the environment, and includes items that describe distraction (for example: When you feel the urge to eat sweets, try to find something else to do) and suppression (for example: If you want to eat unhealthy things, say “no” to yourself). The third approach, maintenance of healthy eating goals, reflects strategies to deal with the goal of eating healthily and includes items that describe goal setting and rules (e.g.: Having an agreement with yourself about how many sweets you can have a day) and reflection on goals (for example: If I am eating too much, I think about how this behaviour can impair the practice of physical exercise). In the theoretical model, the three approaches then represent one-third-order factor that is the self-regulatory competence. In the present study, the omega coefficients for the six specific self-regulation strategies were the follows: for avoidance of temptations it was 0.60, for controlling temptations it was 0.60, for distraction it was 0.72, for suppression it was 0.78, for goal and rule setting it was 0.76, and finally for goal deliberation it was 0.74.

#### Eating self-efficacy brief scale

*Eating Self-Efficacy Brief Scale* (ESEBS, Lombardo et al., 2021) measures confidence in one’s ability to self-regulate eating in situations where people face social or emotional pressures due to excessive food intake. The instrument consists of two subscales of four items each (six-point Likert scale from 0 to 5) whereby the person is asked how easy (from “not at all easy” to “completely easy”) it was to resist the urge to eat in situations that challenge the self-regulation of this behaviour, such as social facilitation (e.g., in the presence of other family members and availability of food) or emotional activation (especially negative emotions). In the present study, the omega coefficient for the social self-efficacy subscale was 0.67, while for the emotional self-efficacy subscale it was 0.65.

#### Emotional eating scale

*Emotional Eating Scale* (EES, Arnow et al., 1995); It. Valid. Lombardo and San Martini (2005) include 25 items assessing the desire to eat after negative emotions and it is composed of three subscales: emotional eating after depression (EES-D), emotional eating after anxiety/confusion (EES-A), and emotional eating after anger (EES-R). Participants are asked to indicate the extent to which certain feelings lead them to feel an urge to eat (e.g., when they felt depressed, bored, angry, agitated, etc.) on a five-point scale ranging from 1 (“no desire to eat”) to 5 (“an overwhelming urge to eat”). In the present study, the coefficient omega for the EES-D subscale was 0.79, for the EES-A, it was 0.70, finally for the EES-R it was 0.76.

#### Scale of attitudes towards healthy eating

*Scale of Attitudes towards Healthy Eating* (SAHE, Mullan et al., 2013) assesses three dimensions of attitude through six items for each different domain, with responses provided on a semantic differential scale described by the bipolar adjectives: ‘bad–good’, ‘harmful–beneficial’, ‘unenjoyable–enjoyable’, ‘useful–useless’, ‘foolish-wise’, and ‘unpleasant-pleasant’, in response to a common stem for each scale: “I think eating five portions of fruit and vegetables every day/eating breakfast every day/restricting the consumption of snacks for the next 3 months, is…” Participants were asked to rate their attitudes towards healthy eating on a scale from 1 to 7, with a higher score indicating a more positive attitude. In the present study, the coefficient omega for the attitude towards eating five portions of fruit and vegetables every day was 0.74, for the attitude towards eating breakfast every day it was 0.73 and for the attitude towards restricting the consumption of snacks for the next 3 months it was 0.88.

#### Body mass index

Self-reported height and weight were used to calculate *Body Mass Index* (BMI).

### Analysis

There were no missing data in the present database, because the online survey was set up with mandatory answers to all questions.

The posited model with six specific self-regulation strategies (first-order factors) loaded on three general self-regulation approaches (second-order factors: addressing the temptation directly, addressing the psychological meaning of temptation, and addressing the goal directly) which in turn are assumed to represent one higher-order factor (third-order factor: the self-regulatory competence). The goodness-of-fit of the model with the data was assessed using the maximum likelihood robust (MLR) chi-square test statistic and multiple fit indices: the Comparative Fit Index (CFI) that is expected to be ≥0.90 in an acceptable model fit; the Root Mean Square Error of Approximation (RMSEA) and the Standardized Root Mean Square Residual (SRMR), that are expected to be ≤0.05 in an acceptable fit (Hu and Bentler, 1999; Kaplan, 2000; Schreiber et al., 2006). We preferred to use robust ML estimation (MLR) instead of WLSMV because previous studies have suggested to choose the MLR estimation when the number of response categories is equal or greater than 5 (Rigdon, 1998; Raykov, 2012; Morin et al., 2020) and this was the case in the present study.

The measurement invariance of the TESQ-E across gender was examined by means of a hierarchical series of multigroup confirmatory factor analyses (CFAs), imposing increasingly restrictive equality constraints on the model’s parameters in accordance with the general procedures suggested by Chen et al. (2005) for higher-order factor models. Firstly, we assessed configural invariance, by specifying an unrestricted baseline model (Model 1) in which each group had the same structure (Chen et al., 2005). Secondly, we tested factor loading invariance for first-, second-, and third-order factors (Model 2, 3, and 4) to assess metric invariance. In Model 2, all the first-order factor loadings were constrained to be equal across groups. In Model 3, second-order factor loadings were also constrained to be equal across groups, while in Model 4 third-order factor loadings constraints were added. Model 5, 6, and 7 imposed additional constraints: in Model 5 intercepts of the measured variables are constrained to be equal across groups, in addition to factor loadings of the latent variables (Model 4). In Model 6, intercepts of the measured variables as well as of first-order factors were constrained to be equal across groups, in Model 7, a constraint to the intercepts of second-order latent factors was added. In each step of the analysis, we compared the fit of the nested models by using several indices and according to Chen (2007) recommendations. To test loading invariance, a change <0.010 in CFI, along with a change of <0.015 in RMSEA or of <0.030 in SRMR would indicate invariance, while for testing intercept invariance a change of <0.015 in RMSEA or of <0.010 in SRMR would suggest measurement invariance.

The latent mean differences across gender were estimated for the six specific self-regulation strategies.

Furthermore, the relationships between the six self-regulation strategies and several related self-regulated eating constructs were analysed to support the convergent validity of the scale. The Pearson’s correlation was computed between the mean scores of the six self-regulation strategies and the mean scores of the following scales: the Eating Self-Efficacy Brief Scale (ESEBS; Lombardo et al., 2021), the Emotional Eating Scale (EES; Arnow et al., 1995; Lombardo and San Martini, 2005), and the attitudes towards three of the most-recognized healthy eating behaviours (Ajzen, 1991; Wong and Mullan, 2009; Mullan et al., 2013; Girelli et al., 2016a). Finally, the correlation between the six self-regulation strategies and BMI was also calculated.

Preliminary analyses were performed using IBM SPSS Statistics version 27 (IBM Corp. Released, 2020), while all the other analyses were carried out using Mplus 8 software, version 1.6 (Muthén and Muthén, 2017).

## Results

### Descriptive statistics

Firstly, we decided to remove the items with skewness greater than 2 or less than 2 and to remove the items with a kurtosis greater than 2 or less than-2 (three items). Problems with non-normally distributed data could be related to socio-cultural characteristics of the context where the study has been conducted. Subsequently, a fourth item was removed for the same reason. These steps were aimed at selecting the best items for each dimension, based on both empirical and theoretical assumptions and they resulted in a selection of three items for avoidance of temptations, three items for controlling temptations, three items for suppression, and three items for goal and rule settings. The means, SDs, skewness, and kurtosis of the 20 selected items are presented in Table 1.

**Table 1:** Means, SDs, skewness, and kurtosis of the selected items.

	Means	*SD*	Skewness	Kurtosis
Approach: Addressing the temptation directly					*Strategies:* *Avoidance of temptations*				
Item 1	2.21	1.21	0.81	−0.31
Item 7	2.11	1.24	0.91	−0.26
Item 13	2.51	1.18	0.61	−0.50
*Controlling temptations*				
Item 2	2.54	1.34	0.53	−0.94
Item 8	2.41	1.13	0.67	−0.40
Item 14	3.10	1.22	0.13	−1.09	Approach: Addressing the psychological meaning of temptation					*Strategies:* *Distraction*				
Item 3	2.30	1.32	0.71	−0.72
Item 9	2.19	1.17	0.94	0.05
Item 15	2.85	1.23	0.24	−0.95
Item 19	1.91	1.11	1.19	0.64
*Suppression*				
Item 4	2.18	1.19	0.88	−0.15
Item 10	1.70	1.03	1.52	1.53
Item 16	2.72	1.23	0.34	−0.89	Approach: Addressing the goal directly					*Strategies:* *Goal and rule setting*				
Item 5	2.04	1.05	1.08	0.67
Item 11	2.74	1.26	0.38	−0.94
Item 17	2.38	1.23	0.69	−0.50
*Goal deliberation*				
Item 6	2.14	1.40	0.95	−0.49
Item 12	2.29	1.17	0.74	−0.32
Item 18	2.09	1.23	0.97	−0.13
Item 20	2.66	1.24	0.41	−0.86

*N* = 645.

### Factorial structure of the TESQ-E

**Figure 1:**
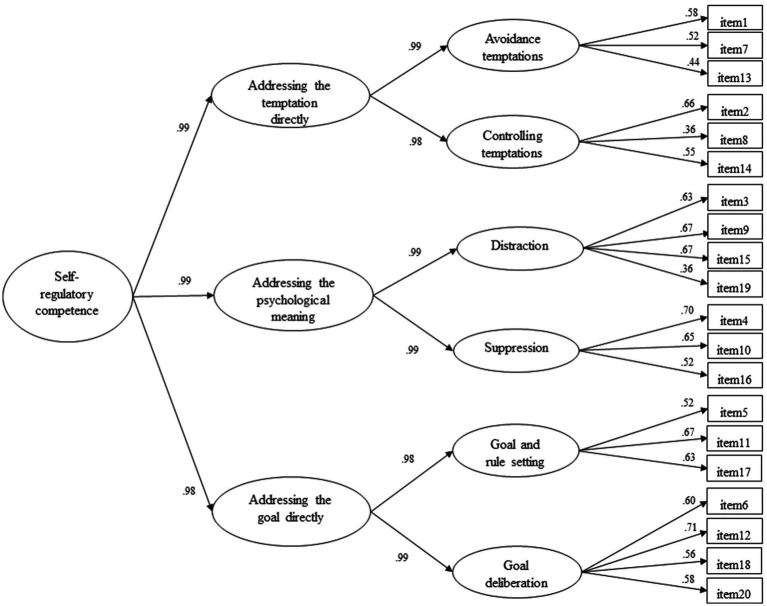
Confirmatory factor analysis results. All the estimates are standardized. All the estimates are statistically significant *p*  <  0.001.

The results of the confirmatory factor analysis (CFA) provided support for the posited model for the TESQ-E [χ^2^ (152) = 336.480, *p* < 0.001; CFI = 0.95; RMSEA = 0.04; SRMR = 0.04]. Except for the chi-square test (probably affected by the large size of the sample used in the present study), all the fit indices indicated a good fit of the model with the empirical data (Hu and Bentler, 1999; Schreiber et al., 2006). The results confirmed the hypothesised factorial structure: six specific self-regulation strategies (first-order factors) loaded on three general self-regulation approaches (second-order factors), which in turn described an overall self-regulatory competence (third-order factor). The standardised factor loadings are presented in Figure 1. All the loadings were statistically significant (*p* < 0.001).

### Measurement invariance across gender of third-order factor model of TESQ-E

The results provided support for the posited model for males [χ^2^ (152) = 248.902, *p* < 0.001; CFI = 0.94; RMSEA = 0.05; SRMR = 0.05] as well as for females [χ^2^ (152) = 284.621, *p* < 0.001; CFI = 0.93; RMSEA = 0.05; SRMR = 0.05]. To test whether the third-order factor structure is statistically equivalent across boys and girls, a hierarchical series of nested models were tested, following the general procedures suggested by Chen et al. (2005). The fit statistics for each model are presented in Table 2. Model 1 showed good fit indices and provided support for the configural invariance of the TESQ-E across gender. The comparison of the fit for the different nested models showed that the difference in the CFI between the models were smaller than the cut-off criterion (Model 2 vs. Model 1 ΔCFI = 0.005, ΔRMSEA = 0.001, and ΔSRMR = 0.011; Model 3 vs. Model 2 ΔCFI = 0.001, ΔRMSEA = 0.000, and ΔSRMR = 0.001; and Model 4 vs. Model 3 ΔCFI = 0.000, ΔRMSEA = 0.000, and ΔSRMR = 0.000). This indicated that the first, second and third-order factor loadings were invariant across males and females thus confirming the metric invariance of the TESQ-E. Finally, the comparison of the metric invariance model (Model 4) with models with increasing constraints in the intercepts of the measured variables (Model 5) and of first-order (Model 6) and second-order factors (Model 7) provided support for the scalar invariance of the TESQ-E (Model 5 vs. Model 4 ΔCFI = 0.006, ΔRMSEA = 0.001, ΔSRMR = 0.000; Model 6 vs. Model 5 ΔCFI = 0.001, ΔRMSEA = 0.000, ΔSRMR = 0.000; and Model 7 vs. Model 6 ΔCFI = 0.001, ΔRMSEA = 0.000, ΔSRMR = 0.001). The overall results provided support for the full scalar invariance of the scale across gender, thereby allowing to compare the factor means of the TESQ-E across groups.

**Table 2:** Summary of fit statistics for testing measurement invariance across gender of third-order factor model of Tempest Self-Regulation Questionnaire for Eating (TESQ-E).

	χ^2^	*df*	RMSEA	SRMR	CFI	Model comparison	ΔCFI	ΔRMSEA	ΔSRMR
*Model 0*									
Model fit males	248.902	152	0.050	0.050	0.939				
Model fit females	284.621	152	0.050	0.050	0.931				
*Model 1*									
Configural invariance	549.178	313	0.048	0.050	0.932				
*Model 2*									
First-order factor loadings invariant	581.650	327	0.049	0.061	0.927	Model 2 vs. Model 1	0.005	0.001	0.011
*Model 3*									
First-and second-order factor loadings invariant	588.046	330	0.049	0.062	0.926	Model 3 vs. Model 2	0.001	0.000	0.001
*Model 4*									
First-, second-and third-order factor loadings invariant	590.177	332	0.049	0.062	0.926	Model 4 vs. Model 3	0.000	0.000	0.000
*Model 5*									
First-, second-and third-order factor loadings and intercept of measured variables invariant	624.697	346	0.050	0.062	0.920	Model 5 vs. Model 4	0.006	0.001	0.000
*Model 6*									
First-, second-and third-order factor loadings and intercept of measured variables and first-order factors invariant	632.476	349	0.050	0.062	0.919	Model 6 vs. Model 5	0.001	0.000	0.000
*Model 7*									
First-, second-and third-order factor loadings and intercept of measured variables, first-order and second-order factors invariant	639.599	352	0.050	0.063	0.918	Model 7 vs. Model 6	0.001	0.000	0.001

### Latent mean differences

Females showed higher levels of goal and rule setting compared to males (0.31, *p* < 0.01) while there were no significant differences across gender in the other five specific self-regulated strategies (Table 3). Therefore, females appear to prefer a strategy that directly addresses the goal by expressing explicit intentions or plans to eat in a healthy way.

**Table 3:** Results of the latent mean differences tests across genders for the six specific self-regulation strategies.

	Gender^a^
Avoidance temptations	0.16
Controlling temptations	0.02
Distraction	0.08
Suppression	0.02
Goal and rule setting	0.31^**^
Goal deliberation	0.11

a Males are the reference group for the comparison (the latent mean for this group is fixed to be zero).

** *p* < 0.01.

### Relationships between the six self-regulation strategies and other self-regulated eating constructs

In order to evaluate the convergent validity of the TESQ-E a new sample of 335 young people (80% females, M_age_ = 25.04; *SD*_age_ = 4.39; min_age_ = 20; max_age_ = 40) was enrolled. Table 4 presents the correlations between the six TESQ-E specific self-regulated strategies and the related eating constructs.

**Table 4:** Correlations between the six TESQ-E subscales and the other self-regulated eating constructs.

		Eating Self-Efficacy (ESEBS)		Emotional Eating (EES)			Healthy eating attitudes (SAHE)			BMI
		Social circumstances	Emotional circumstances	Depression	Anger	Anxiety	Fruit and vegetables	Breakfast	Snacks	
Specific self-regulation strategies	Avoidance of temptations	0.17^**^	−0.00	−0.03	−0.09	−0.04	0.23^***^	0.15^**^	0.15^**^	0.07
	Controlling temptations	0.16^**^	0.10	−0.04	−0.06	−0.06	0.15^**^	−0.04	0.08	0.10
	Distraction	0.06	−0.06	0.05	0.07	0.07	0.10	−0.06	0.04	0.13^*^
	Suppression	0.12^*^	−0.11^*^	0.10	0.02	0.04	0.09	0.01	0.03	0.16^**^
	Goal and rule setting	0.10	−0.04	−0.04	−0.07	−05	0.14^*^	0.07	0.15^**^	0.12^*^
	Goal deliberation	0.09	−0.07	0.11^*^	0.13^*^	0.09	0.12^*^	−0.13^*^	0.02	0.15^**^

BMI, Body Mass Index.

*** *p* < 0.001;

** *p* < 0.01;

* *p* < 0.05.

#### Eating self-efficacy

Both approaches of addressing the temptation directly (avoidance and controlling temptations) were significantly and positively related to self-efficacy in social circumstances, but they are not related to self-efficacy in emotional circumstances. Suppression behaviour is positively associated with self-efficacy in social circumstances while it is negatively related to self-efficacy in emotional situations.

#### Emotional eating

We found that only the goal deliberation strategy was associated with the desire to eat after negative emotions. Specifically, this self-regulated strategy was positively related to both emotional eating after depression and anger.

#### Healthy eating attitudes

Avoidance of temptations was significantly and positively related to the attitudes towards all three healthy eating behaviours (fruit and vegetables, having breakfast, and avoiding high-calorie snacks), while the controlling temptations strategy was only significantly positively related to the positive attitude towards the consumption of fruits and vegetables. Finally, the strategies of addressing the goal directly (goal and rule setting and goal deliberation) appeared to be related to the attitudes towards healthy eating: both strategies were positively associated with the positive attitudes towards the consumption of fruits and vegetables; moreover, goal and rule setting was also associated with the positive attitudes towards avoiding high-calories snacks, while goal deliberation was associated with the positive attitudes towards having breakfast every day.

#### Body mass index

Distraction and suppression strategies were both positively and significantly related to the BMI. The same pattern of correlation has been shown as regards BMI and goal and rule setting and goal deliberation.

The English and Italian versions of the TESQ-E, as well as the grouping of the items on corresponding factors, are presented in following Table 5.

**Table 5:** Self-Regulation Questionnaire for Eating (TESQ-E).

*Self-Regulation Questionnaire for Eating (TESQ-E)*	
**English version**	**Italian version**
**Instructions:** Thinking about the last 2 weeks, please indicate from 1 (never) to 5 (always) how often you perform the actions described in the following situations.	**Istruzioni:** Pensando alle ultime due settimane, per favore indica da 1 (mai) a 5 (sempre) con quale frequenza metti in atto le azioni descritte nelle seguenti situazioni.
1. If I pass a bakery, I avoid looking at display in the window (AV).	1. Se passo davanti una panetteria, evito di guardare nella vetrina espositiva.
2. If I am watching TV, I make sure that the crisps are out of reach (CO).	2. Se sto guardando la TV, mi assicuro che le patatine siano fuori portata.
3. If I feel tempted to buy candies, I distract myself (DI).	3. Se sono tentato di comprare delle caramelle o dolciumi, cerco di distrarmi.
4. If I pass a bakery, I ignore the smells of tasty foods (SU).	4. Se passo davanti una panetteria o una pasticceria, ignoro i profumi che provengono dall’interno.
5. If I am away from home, I generally plan to bring fruit as a snack (GRS).	5. Se mi trovo fuori casa, generalmente pianifico di portare della frutta per merenda o come spuntino.
6. If I want to have a snack, I try to realize that snacks are bad for your health (GD).	6. Quando voglio fare uno spuntino, cerco di ricordarmi che gli spuntini fanno male alla salute.
7. If I go to the supermarket, I avoid the candy department (AV).	7. Se vado al supermercato, evito lo scaffale con i dolci e le caramelle.
8. If I am behind the PC, I make sure there is some healthy food within reach (CO).	8. Quando sono davanti al PC, faccio in modo di avere del cibo sano a portata di mano.
9. If I feel like eating something, I call a friend instead (DI).	9. Se mi viene voglia di mangiare qualcosa, telefono ad un amico/a invece di assecondare questa tentazione.
10. I use willpower to stay away from unhealthy snacks (SU).	10. Io uso la forza di volontà per stare lontano dagli spuntini malsani.
11. I have an agreement with myself about how many candies I can have per day (GRS).	11 Ho fatto un patto con me stesso sul numero massimo di dolci o caramelle da consumare in un giorno.
12. If I think I may be overeating, I think of how this may compromise exercising (GD).	12. Se penso che stia esagerando nel mangiare, mi focalizzo su come questo possa compromettere il mio allenamento.
13. If I am bored, I stay away from the kitchen (AV).	13. Quando mi annoio rimango volutamente lontano dalla cucina.
14. If I want to eat candy, I take a few and put the rest of the bag away (CO).	14. Se ho voglia di mangiare dolciumi o caramelle, ne prendo un po’ e metto *via* il resto.
15. If I am getting hungry before dinner, I try to keep myself busy (DI).	15. Se mi viene fame prima di cena, cerco di tenermi occupato/a.
16. If I go to a party with lots of snacks, I ignore the food (SU).	16. Se vado a una festa dove vi sono molti snack a disposizione, ignoro il cibo.
17. I set goals to eat healthy for myself (GRS).	17. Fisso degli obiettivi per riuscire a mangiare in modo salutare.
18. If I want to take a snack, I remember that I want to stay attractive (GD).	18. Quando ho voglia di fare uno spuntino, ricordo a me stesso/a che voglio rimanere attraente.
19. If I have the urge to eat candy, I find something else to do (DI).	19. Se avverto l’urgenza di mangiare caramelle o dolci, mi trovo qualcos’altro da fare.
20. If I feel like eating something unhealthy, I think about whether I really want it (GD).	20. Quando mi va di mangiare qualcosa di poco sano, penso se lo voglio veramente.

AV, Avoidance Temptations; CO, Controlling Temptations; DI, Distraction; SU, Suppression; GRS, Goal and Rule Setting; and GD, Goal Deliberation.

## Discussion

The TESQ-E is an instrument that investigates individuals’ self-regulation and self-control strategies to counteract the desire and temptation to eat unhealthy foods and to choose healthy foods (De Vet et al., 2014). The questionnaire was initially validated in a sample of adolescents across nine European countries (Belgium, Denmark, Finland, Germany, Poland, Portugal, Romania, The Netherlands, and the United Kingdom). A previous validation study with a single country sample has been conducted solely in Portugal (Gaspar et al., 2015). However, until now, no study had yet validated the scale in the Italian context, moreover, no study has investigated the psychometric properties of the scale in a sample of young adults, so the present contribution certainly fills a gap in the Italian psychometric landscape. The tool allows the identification of strategies used by young adults to self-regulate and control themselves, which is a fundamental skill for the adoption of healthy eating behaviours. Finally, the studies conducted so far did not examine measurement invariance of the scale across gender, which is important, considering the differences in eating behaviour patterns among males and females.

The posited model demonstrated a good fit to the data, showing six specific self-regulation strategies loading on three general self-regulation approaches (addressing the temptation directly, addressing the psychological meaning of temptation, and addressing the goal directly) which represented one higher-order factor, the self-regulatory competence. This structure is consistent with validation studies conducted in other cultures (De Vet et al., 2014).

The third-order factor structure of the scale showed the scalar measurement invariance across gender. Males and females, therefore, appear to conceptualize the different forms of the self-regulatory competence—and thus to interpret the corresponding items—in a very similar way. To our knowledge, this is the first time that the measurement invariance of this instrument has been tested and proven and this allows us to use the TESQ-E scores to meaningfully compare the different self-regulation strategies across gender. Latent means differences showed that female participants of this study exhibited a higher level of self-regulatory strategy related to directly addresses the goal by expressing explicit intentions or plans to eat in a healthy way, thus confirming previous studies that have shown differences across gender in the adoption of regulation strategies (i.e., Anderson et al., 2007; Gaspar et al., 2015).

Concerning the correlations between the six self-regulated strategies and several other self-regulated eating constructs, we found positive associations between strategies that directly addressed temptations (avoidance and controlling) and those addressing the meaning of temptations (suppression) with perceived eating self-efficacy in social circumstances (e.g., Alivernini and Manganelli, 2016), which refers to the individuals’ sense of effectiveness in dealing with external pressures for excessive food intake, similar to avoidance/controlling of temptations and suppression (Shieh et al., 2015). With respect to healthy eating attitudes, the strongest correlations and greater number of correlations with different strategies were observed for the attitudes towards fruit and vegetable consumption: young adults who reported to engage in self-regulation strategies to a greater extent had more positive attitudes towards the consumption of fruits and vegetables. This is relevant because fruit and vegetable consumption is known to be an important contributor to preventing excess weight and obesity, especially in young adults (Pem and Jeewon, 2015). Self-regulation strategies were also associated with the attitudes towards two other eating behaviours, such as breakfast consumption and avoiding the consumption of snacks. The latter two behaviours may be slightly more dependent on environmental factors, and therefore less susceptible to self-regulation by young adults (i.e., DeJong et al., 2009). The fact that almost none of the self-regulatory strategies were associated with emotional eating may suggest that employing these strategies does not depend on coping with negative affect by eating even though they involve active self-regulation (Evers et al., 2010). However, our results also showed that some correlations were low or non-significant, namely, contrary to what might have been expected, no associations emerged between the strategies included in the two second-order approaches (addressing psychological meaning and addressing goal) with the measure of self-efficacy in regulating eating behaviour in situations involving the co-presence of other people. It would seem, for those who participated in the study, more natural to associate the perception of self-efficacy in such situations with the implementation of strategies of direct reaction to temptation (avoidance or controlling) that allow them to immediately notice its effects (according to an if-then plan; Churchill et al., 2019; Bieleke et al., 2021; Chami et al., 2022), rather than engaging in general actions of manipulating/occulting the meanings associated with food or imposing rules of restraint by first setting goals or standards of reference (Hommel, 2022). On the explanatory level, specifically cultural elements rather refractory to rigid dietary control (Medina, 2021) could be implicated for such evidence, or more simply the lack yet in this age group of a behavioural orientation inspired and consciously guided by a systematic eating plan, prolonged over time and supported by general rules or principles of reference (Ripabelli et al., 2017; Diotaiuti et al., 2021a).

As reported by several studies, self-control over eating can be particularly weak at times of increased psychological tension and distress, including collective distress, as may have occurred during the current pandemic, where social constraints have also often reinforced sedentary lifestyles in young adults, fostering unregulated and compensatory eating behaviors (Diotaiuti et al., 2021b; Cavicchiolo et al., 2022; Ferrara et al., 2022; Galli et al., 2022), and even addictive practices (Petruccelli et al., 2014; Avena et al., 2021; Lim, 2021; Diotaiuti et al., 2022).

An extensive body of literature has demonstrated that self-regulation and self-control strategies in young adults can help people to adopt health-related behaviour (e.g., Girelli et al., 2020; Mallia et al., 2020), especially in the eating domain (e.g., Van Duyn and Pivonka, 2000; Wills et al., 2007; Gerrits et al., 2010) and we believe that the present study has made a significant contribution to this matter by expanding to a different context the range of instruments available for investigating eating self-regulation strategies.

## Limitations

It is important to acknowledge some important limitations of the study. First, the findings only relied on the use of self-reported measures. Future studies could consider including more objective measures of self-regulation or other related constructs. Second, omega coefficients for the factors of avoidance of temptations and controlling temptations did not reach the commonly used cut-off criteria. This can possibly depend on the low number of items of the scales. Third, future studies should include other demographic and socio-cultural variables (such as the socioeconomic status of the students), as they might be correlated with self-regulated eating strategies (e.g., de Matos et al., 2016) and should further investigate convergent validity of the scale with related constructs. Finally, only Italian data were considered. Future research should therefore be conducted to generalize our findings across various other countries and cultural contexts.

## Conclusion

The results of this study conducted on a sample of Italian young adults confirmed that the *Self-Regulation Questionnaire for Eating* (TESQ-E) is a psychometrically sound measure that can be effectively used to measure self-regulation eating strategies in young adults. Moreover, it encompasses the complex and multifaceted nature of self-regulation and self-control strategies by considering a third-factor structure of the scale. According to Fishbach and Converse (2011), self-regulation strategies can be grouped into three general self-regulation approaches which form one overarching concept of self-regulation. The present study supports this claim, adding further evidence to the literature of the good psychometric properties, validity, and broad applicability of this theory-based instrument. Measuring self-regulation eating strategies in young adults may help prevent chronic diseases in adulthood (Ezzati et al., 2004; Allison et al., 2008). In addition, the scale would allow to reliably measure the effects of specific interventions aimed at promoting healthy eating behaviours and preventing unhealthy eating habits (Girelli et al., 2016a,b).

## Data availability statement

The raw data supporting the conclusions of this article will be made available by the authors, without undue reservation.

## Ethics statement

The studies involving human participants were reviewed and approved by Institutional Review Board (IRB) of the University of Cassino and Southern Lazio. The participants provided their written informed consent to participate in this study.

## Author contributions

PD, SM, and GV designed the study. PD, EC, LG, and SM analysed the data and discussed the results. PD, EC, and LG drafted the manuscript. FM, FB, and GV revised the manuscript. All authors contributed to the article and approved the submitted version.

## Conflict of interest

The authors declare that the research was conducted in the absence of any commercial or financial relationships that could be construed as a potential conflict of interest.

## Publisher’s note

All claims expressed in this article are solely those of the authors and do not necessarily represent those of their affiliated organizations, or those of the publisher, the editors and the reviewers. Any product that may be evaluated in this article, or claim that may be made by its manufacturer, is not guaranteed or endorsed by the publisher.

## Footnotes

^1^ http://www.tempestproject.eu/
